# Supplementary material for: An improved neuroanatomical model of the default-mode network reconciles previous neuroimaging and neuropathological findings
Source: Commun Biol. 2019 Oct 10;2:370. doi: 10.1038/s42003-019-0611-3 (PMC6787009; doi:10.1038/s42003-019-0611-3)
Supplement: Supplementary file 1 — Supplementary Figures and Tables [file 42003_2019_611_MOESM1_ESM.pdf]

- Supplementary Figures

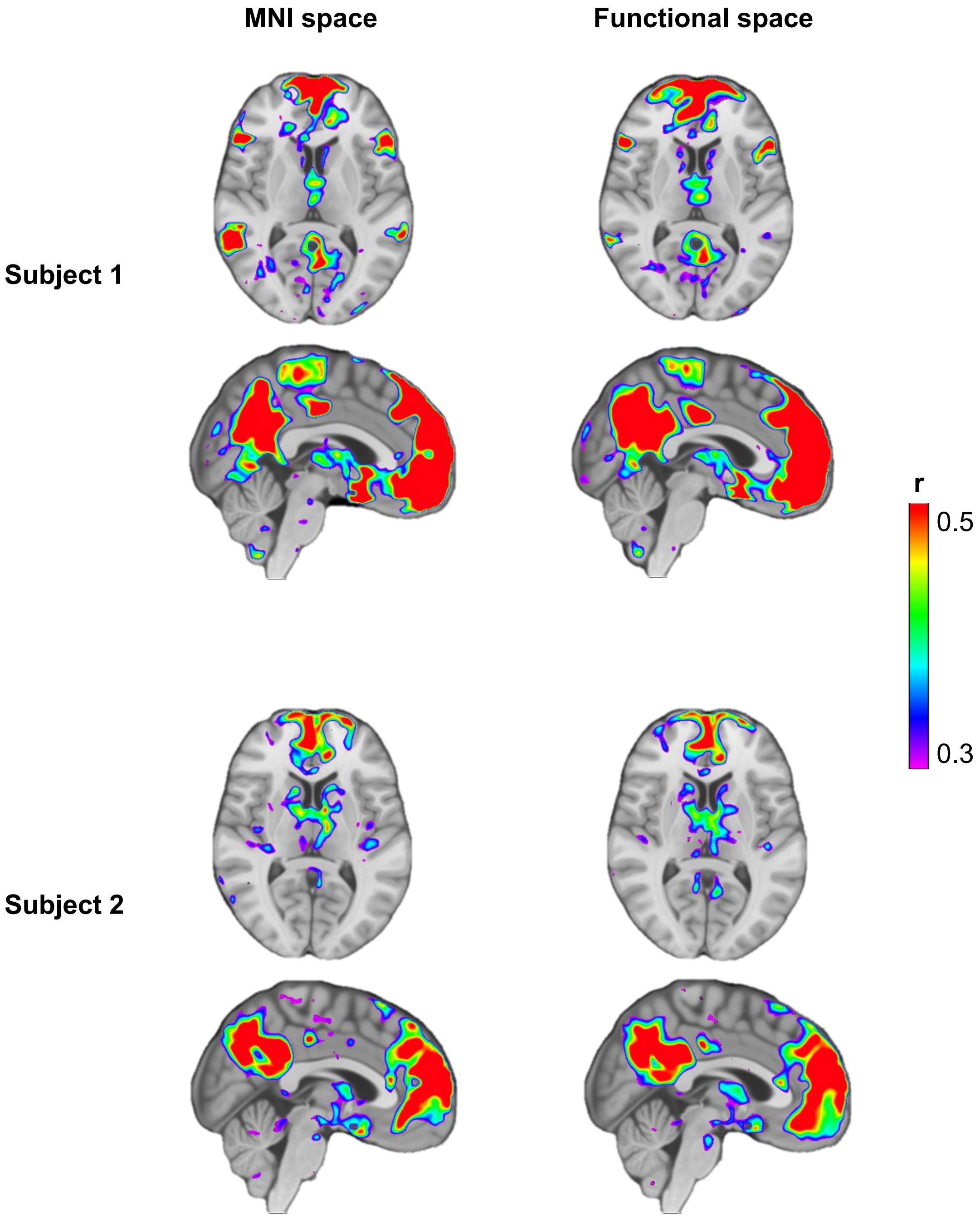

Supplementary Figure 1. Two representative cases of individual DMN maps in the MNI152 space (left column) and in the functional space (right column). Colour maps represent Pearson's correlation values.

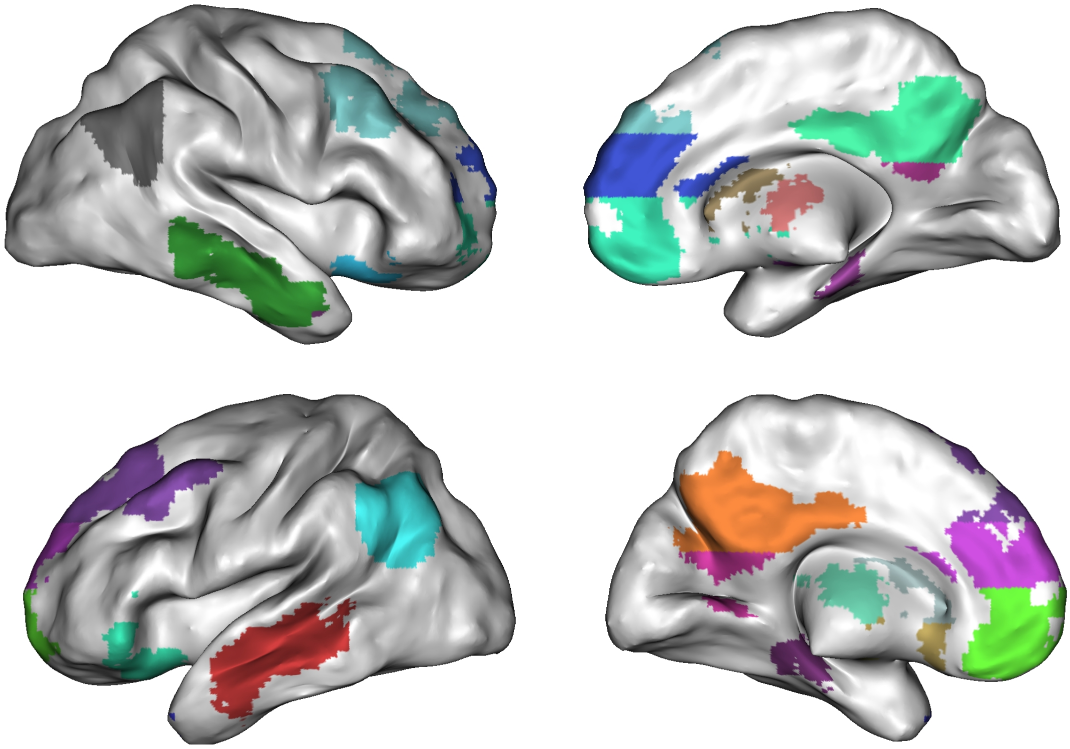

Supplementary Figure 2. DMN regions of interest used for the functional and for the structural connectivity post-hoc analyses represented on an inflated brain surface. This map includes the subcortical structures uncovered with functional alignment; cerebellar and midbrain regions are not represented. Right hemisphere is represented in the upper images and the left hemisphere in the bottom.

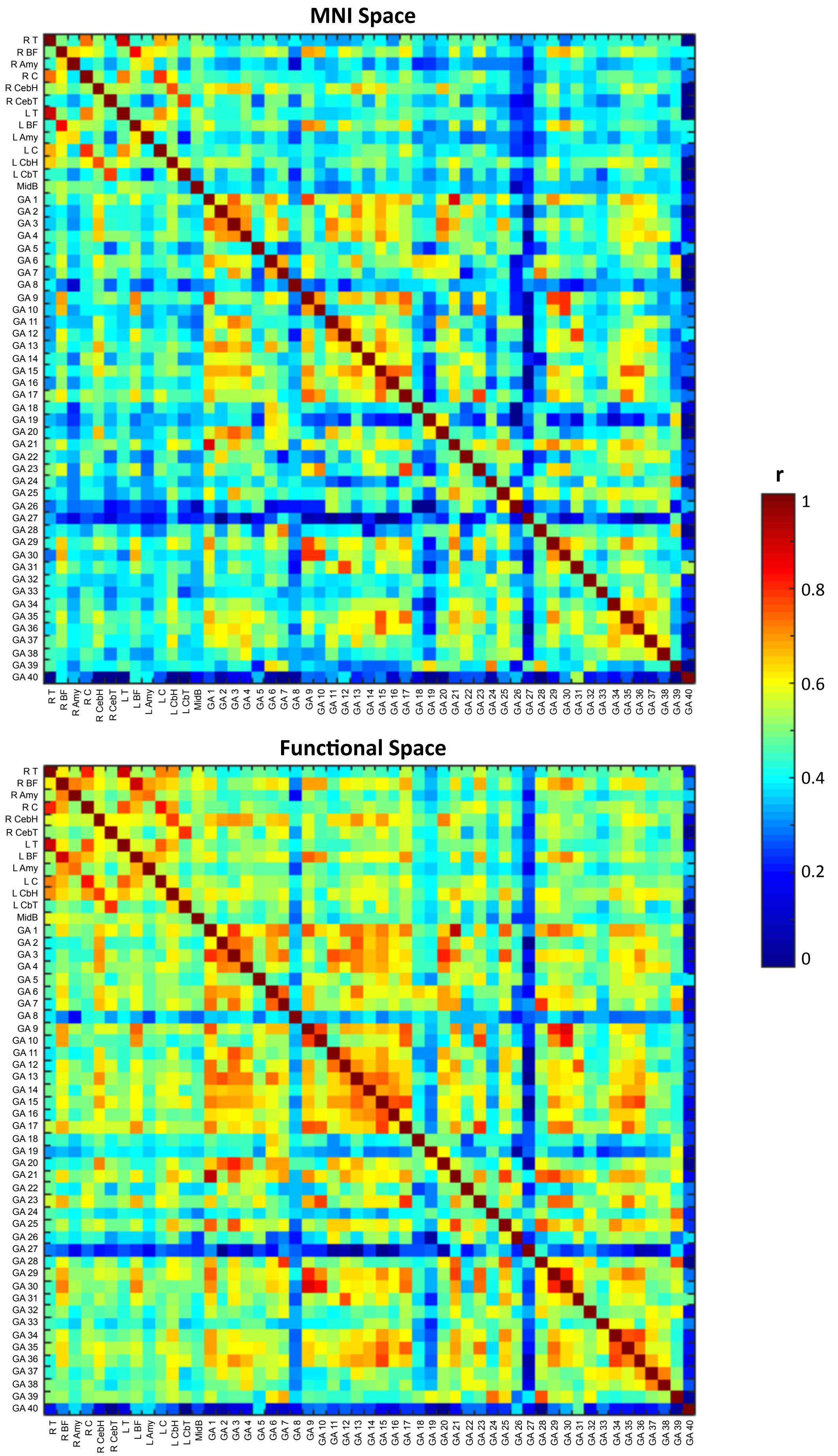
MNI Space
Functional Space
r
1
0.8
0.6
0.4
0.2
0
R T
R BF
R Amy
R C
R CebH
R CebT
L T
L BF
L Amy
L C
L CbH
L CbT
MidB
GA 1
GA 2
GA 3
GA 4
GA 5
GA 6
GA 7
GA 8
GA 9
GA 10
GA 11
GA 12
GA 13
GA 14
GA 15
GA 16
GA 17
GA 18
GA 19
GA 20
GA 21
GA 22
GA 23
GA 24
GA 25
GA 26
GA 27
GA 28
GA 29
GA 30
GA 31
GA 32
GA 33
GA 34
GA 35
GA 36
GA 37
GA 38
GA 39
GA 40

Supplementary Figure 3. Functional connectivity matrices of the Pearson's correlations between rs-*f*MRI time-series of the regions of interest in the structural and in the functional space, using cortical DMN areas according to Gordon et al (2016) instead of the ones defined in classical anatomical models of Andrews-Hanna et al (2010) and Buckner et al (2008). Correlations were higher in the functional space than in the MNI space in 92% of cells, and this difference was statistically significant in 5% of cells (p-value< 0,00004, after Bonferroni's correction for multiple comparisons). R, Right; L, Left; BF, Basal Forebrain; C, Caudate; T, Thalamus; CbH, Cerebellar Hemisphere; CbT, Cerebellar Tonsil; Amy, Amygdala; MidB, Midbrain; GA, Gordon's Area.

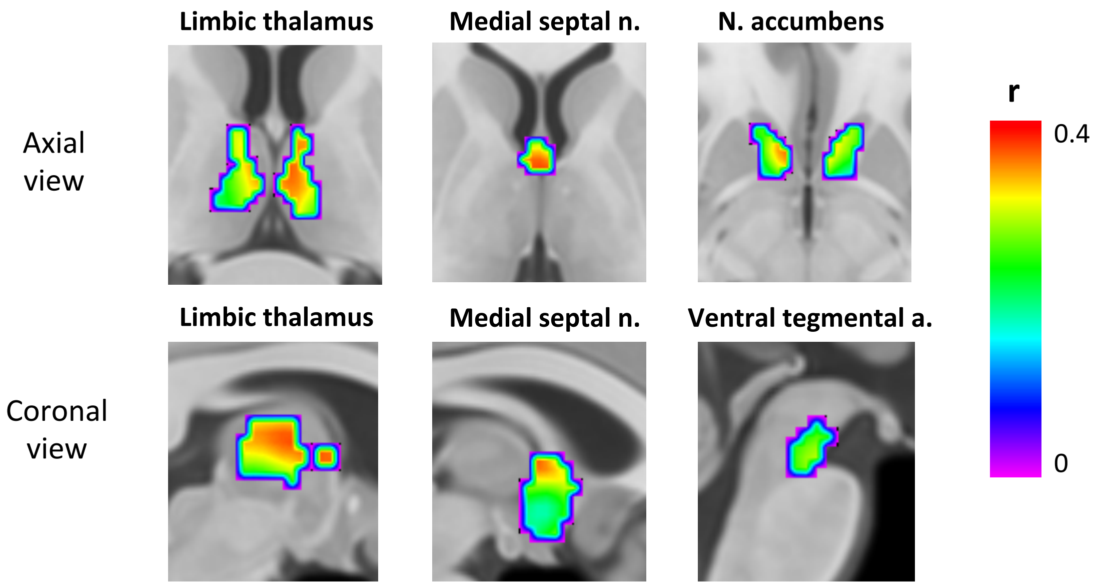

Supplementary Figure 4. Mean correlation values of the functionally-aligned individual DMN networks superimposed in the MNI152 space. Colour bar represents Pearson's correlation.

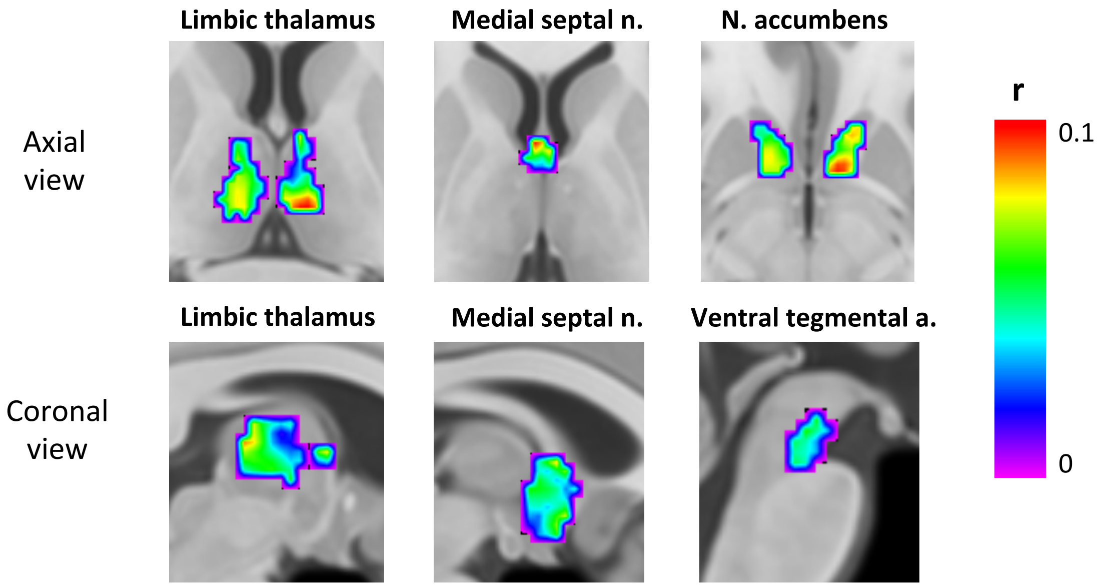

Supplementary Figure 5. Standard deviation of the correlation values of the functionally-aligned individual DMN networks superimposed in the MNI152 space. Colour bar represents Pearson's correlation.

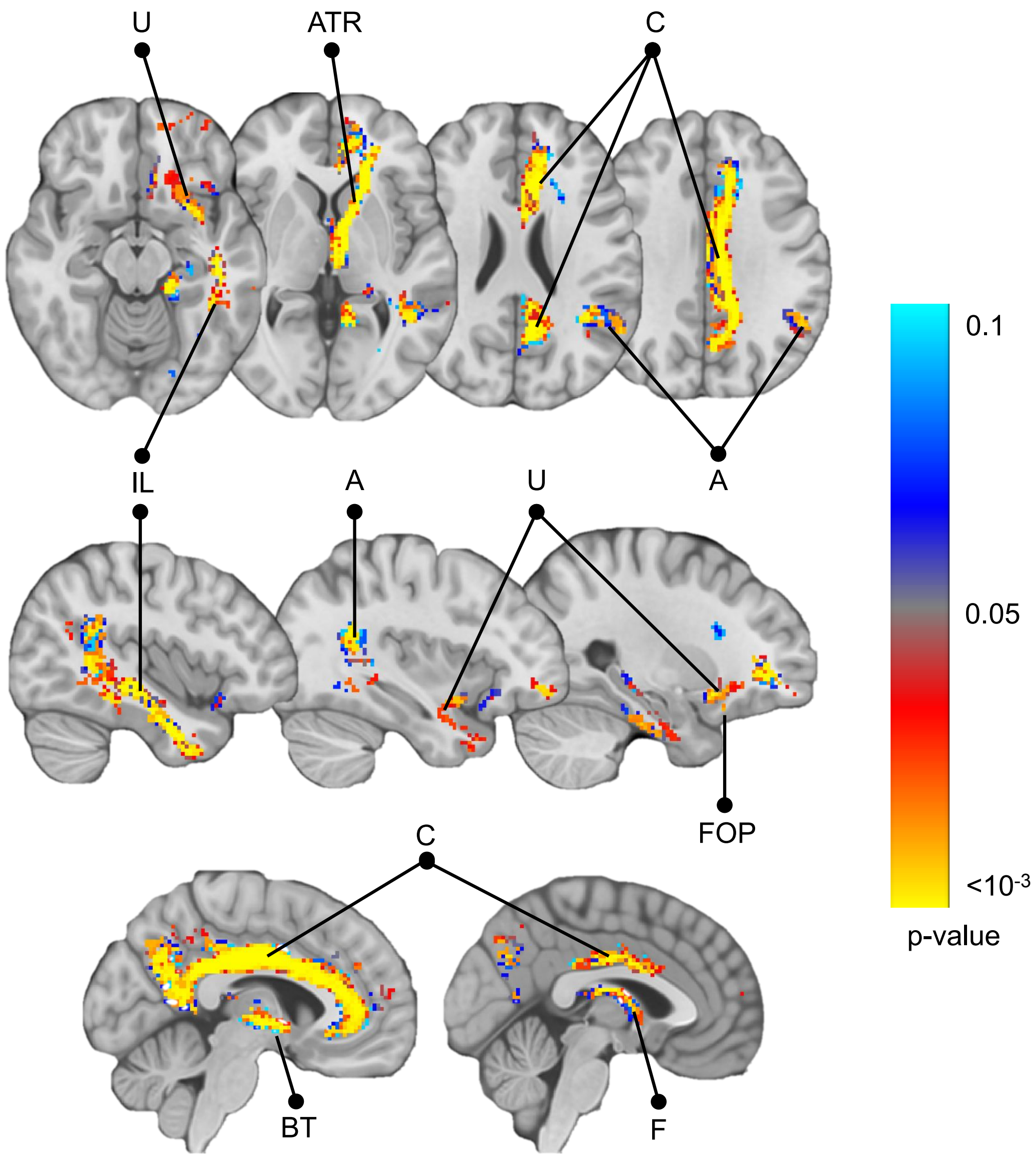

Supplementary Figure 6. Statistical maps of the group representative tracts. Colour bar represents the p-value in each voxel (one sample T-test). A, Arcuate (posterior); ATR, Anterior Thalamic Radiations; BT, Basal forebrain – Thalamus; C, Cingulum; F, Fornix; FOP, Frontal Orbito-Polar; IL, Inferior Longitudinal; U, Uncinate.

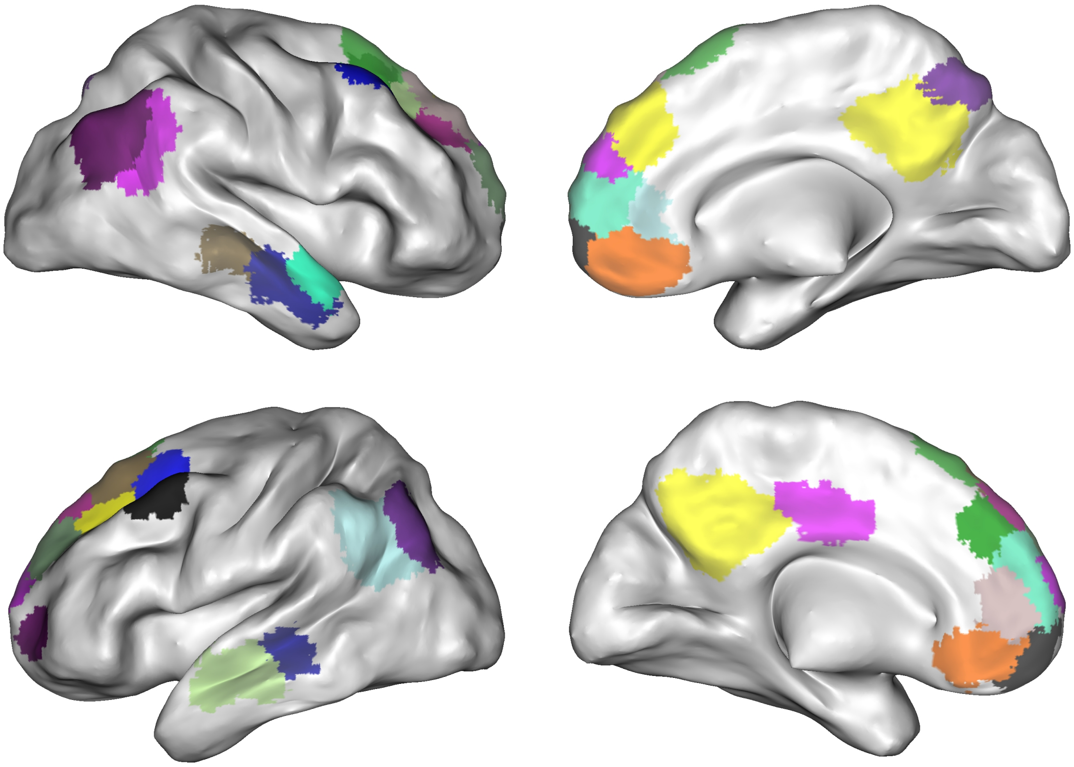

Supplementary Figure 7. DMN nodes according to the functional parcellated cortical template of Gordon and collaborators (2016), represented on an inflated cortical surface. Right hemisphere is represented in the upper images and the left hemisphere in the bottom.

- Supplementary Tables

Supplementary Table 1. Node degree and betweenness centrality of the network nodes. Data is presented as median [interquartile range].

**Nodes**	**Node degree**	**Betweenness centrality**
Right Ventro-Median Prefrontal Cortex	12 [10 – 14]	0.104 [0.087 – 0.126]
Left Ventro-Median Prefrontal Cortex	12 [11 – 14]	0.070 [0.041 – 0.123]
Right Antero-Median Prefrontal Cortex	12 [9 – 13]	0.060 [0.029 – 0.093]
Left Antero-Median Prefrontal Cortex	11 [10 – 12]	0.040 [0.025 – 0.058]
Left Dorsal Prefrontal Cortex	10 [9 – 11]	0.075 [0.055 – 0.095]
Right Dorsal Prefrontal Cortex	10 [8 – 11]	0.057 [0.033 – 0.116]
Left Posterior Cingulate Cortex	9 [8 – 10]	0.025 [0.014 – 0.055]
Left Thalamus	9 [7 – 11]	0.029 [0.016 – 0.047]
Left Basal Forebrain	8 [7 – 9]	0.032 [0.021 – 0.058]
Right Posterior Cingulate Cortex	8 [7 – 10]	0.028 [0.012 – 0.047]
Left Retrosplenial Cortex	8 [7 – 9]	0.020 [0.008 – 0.039]
Right Basal Forebrain	7 [5 – 8]	0.024 [0.011 – 0.054]
Right Retrosplenial Cortex	7 [6 – 8]	0.020 [0.008 – 0.035]
Right Thalamus	7 [5 – 9]	0.033 [0.016 – 0.057]
Left Caudate	5 [4 – 6]	0.003 [0.001 – 0.015]
Left Parahippocampal region	5 [4 – 6]	0.003 [0.001 – 0.042]
Right Middle Temporal Gyrus	5 [3 – 6]	0.020 [0.007 – 0.042]
Left Middle Temporal Gyrus	4 [4 – 6]	0.013 [0.005 – 0.060]
Left Ventro-Lateral Prefrontal Cortex	4 [3 – 4]	0.002 [0.001 – 0.017]
Right Parahippocampal region	4 [3 – 5]	0.004 [0.001 – 0.017]
Left Temporal Pole	3 [2 – 5]	0.002 [0.000 – 0.014]
Right Caudate	3 [2 – 4]	0.001 [0.000 – 0.002]
Left Posterior Parietal Cortex	3 [2 – 3]	0.002 [0.000 – 0.018]
Midbrain	3 [1 – 5]	0.000 [0.000 – 0.059]
Right Cerebellar hemisphere	2 [1 – 3]	0.000 [0.000 – 0.031]
Right Temporal Pole	2 [0 – 5]	0.003 [0.000 – 0.032]
Right Posterior Parietal Cortex	2 [2 – 3]	0.001 [0.000 – 0.005]
Right Ventro-Lateral Prefrontal Cortex	2 [2 – 3]	0.000 [0.000 – 0.002]
Left Amygdala	1 [1 – 2]	0.000 [0.000 – 0.000]
Left Cerebellar Hemisphere	1 [1 – 2]	0.000 [0.000 – 0.004]
Left Tonsil	1 [0 – 1]	0.000 [0.000 – 0.000]

Right Amygdala	1 [0 – 1]	0.000 [0.000 – 0.000]
Right Tonsil	1 [0 – 1]	0.000 [0.000 – 0.000]

Supplementary Table 2. Node degree and betweenness centrality of the network nodes of two representative cases – subjects 1 and 2 (the same subjects represented in the figure 2 and in the table 5).

**Nodes**	**Subject 1**		**Subject 2**	
	Node degree	Betweenness centrality	Node degree	Betweenness centrality
Right Ventro-Median Prefrontal Cortex	12	0,206	9	0,049
Left Ventro-Median Prefrontal Cortex	11	0,072	11	0,064
Right Antero-Median Prefrontal Cortex	8	0,015	12	0,054
Left Antero-Median Prefrontal Cortex	10	0,073	11	0,035
Left Dorsal Prefrontal Cortex	8	0,027	11	0,063
Right Dorsal Prefrontal Cortex	6	0,015	9	0,022
Left Posterior Cingulate Cortex	8	0,032	9	0,039
Left Thalamus	6	0,049	11	0,043
Left Basal Forebrain	5	0,011	10	0,106
Right Posterior Cingulate Cortex	9	0,051	6	0,003
Left Retrosplenial Cortex	7	0,052	7	0,007
Right Basal Forebrain	5	0,022	9	0,133
Right Retrosplenial Cortex	7	0,031	8	0,009
Right Thalamus	2	0,000	12	0,103
Left Caudate	3	0,001	6	0,005
Left Parahippocampal region	2	0,000	4	0,001
Right Middle Temporal Gyrus	2	0,000	1	0,000
Left Middle Temporal Gyrus	8	0,084	5	0,026
Left Ventro-Lateral Prefrontal Cortex	4	0,002	4	0,054
Right Parahippocampal region	2	0,000	4	0,002
Left Temporal Pole	5	0,023	1	0,000
Right Caudate	2	0,000	4	0,006
Left Posterior Parietal Cortex	1	0,000	2	0,009
Midbrain	1	0,000	4	0,002
Right Cerebellar hemisphere	0	0,000	2	0,052
Right Temporal Pole	4	0,093	0	0,000
Right Posterior Parietal Cortex	0	0,000	0	0,000
Right Ventro-Lateral Prefrontal Cortex	2	0,000	1	0,000
Left Amygdala	0	0,000	2	0,000
Left Cerebellar Hemisphere	1	0,000	1	0,000
Left Tonsil	1	0,000	0	0,000
Right Amygdala	0	0,000	0	0,000
Right Tonsil	0	0,000	0	0,000

Supplementary Table 3. MNI coordinates of the centres of gravity and volume of DMN cortical nodes. These nodes, defined by Gordon and collaborators, were used as seeds for building correlation maps. Vol, Volume

**Left hemisphere**					**Right hemisphere**				
**DMN seed**	**MNI (X)**	**MNI (Y)**	**MNI (Z)**	**Vol (mm^3)**	**DMN seed**	**MNI (X)**	**MNI (Y)**	**MNI (Z)**	**Vol (mm^3)**
Seed 1	-7	-50	34	4152	Seed 21	8	-50	34	4096
Seed 2	-12	28	57	1368	Seed 22	12	23	60	1472
Seed 3	-48	-58	32	3240	Seed 23	8	41	4	1136
Seed 4	-5	43	35	1312	Seed 24	23	21	50	968
Seed 5	-3	-15	38	1336	Seed 25	52	-53	28	1736
Seed 6	-20	29	47	1360	Seed 26	61	-26	-6	896
Seed 7	-40	-74	37	2048	Seed 27	9	-68	50	752
Seed 8	-28	55	-1	568	Seed 28	45	-67	35	2776
Seed 9	-6	58	-9	2000	Seed 29	8	63	-5	1800
Seed 10	-7	37	-9	1008	Seed 30	6	44	-13	2104
Seed 11	-60	-28	-9	1248	Seed 31	58	-8	-17	1896
Seed 12	-56	-11	-16	2488	Seed 32	22	33	41	720
Seed 13	-13	48	40	1384	Seed 33	21	45	35	560
Seed 14	-20	53	27	1088	Seed 34	14	62	19	1128
Seed 15	-7	55	19	1936	Seed 35	9	54	13	1232
Seed 16	-14	64	15	904	Seed 36	7	54	30	824
Seed 17	-7	44	7	864	Seed 37	13	45	45	1160
Seed 18	-27	28	39	832	Seed 38	7	41	37	992
Seed 19	-29	15	52	896	Seed 39	31	19	49	840
Seed 20	-39	15	49	1192	Seed 40	53	1	-15	952
