# Supplementary material for: An improved neuroanatomical model of the default-mode network reconciles previous neuroimaging and neuropathological findings
Source: Commun Biol. 2019 Oct 10;2:370. doi: 10.1038/s42003-019-0611-3 (PMC6787009; doi:10.1038/s42003-019-0611-3)
Supplement: Supplementary file 2 — Description of additional supplementary items [file 42003_2019_611_MOESM2_ESM.docx]

**Description of additional supplementary items**

**Supplementary Data 1.** Statistical comparison between the Pearson’s correlations in the functional space and in the MNI space. Each cell represents the corresponding p-value. Significant differences are highlighted (Bonferroni corrected p-value<1E-4).

**Supplementary Data 2.** Median partial correlations between regions of interest, with alignment in the functional space. Partial correlations of the thalamus and of the basal forebrain are highlighted.

**Supplementary Data 3.** Range (max, min; upper right half) and interquartile range (first quartile, third quartile; lower left half) of the partial correlations between regions of interest, with alignment in the functional space. Partial correlations of the thalamus and of the basal forebrain are highlighted.

**Supplementary Data 4.** Statistical comparison between the partial correlations in the functional space and in the MNI space. Each cell represents the corresponding p-value. No significant difference was found for a Bonferroni corrected p-value<0,0001).

**Supplementary Data 5.** Partial correlations between regions of interest of two representative cases, with alignment in the functional space. Subject 1 corresponds to the upper right half and subject 2 to the lower left half (same subjects of the figure 2)
