# Supplementary material for: An improved neuroanatomical model of the default-mode network reconciles previous neuroimaging and neuropathological findings
Source: Commun Biol. 2019 Oct 10;2:370. doi: 10.1038/s42003-019-0611-3 (PMC6787009; doi:10.1038/s42003-019-0611-3)
Supplement: Supplementary file 8 — Reporting Summary [file 42003_2019_611_MOESM8_ESM.pdf]

nature research

Corresponding author(s): Michel Theibaut de Schotten

Last updated by author(s): 2019/07/18

Reporting Summary

Nature Research wishes to improve the reproducibility of the work that we publish. This form provides structure for consistency and transparency in reporting. For further information on Nature Research policies, see Authors & Referees and the Editorial Policy Checklist.

Statistics

For all statistical analyses, confirm that the following items are present in the figure legend, table legend, main text, or Methods section.

n/a	Confirmed	
☐	☒	The exact sample size (*n*) for each experimental group/condition, given as a discrete number and unit of measurement
☒	☐	A statement on whether measurements were taken from distinct samples or whether the same sample was measured repeatedly
☐	☒	The statistical test(s) used AND whether they are one- or two-sided<br>*Only common tests should be described solely by name; describe more complex techniques in the Methods section.*
☐	☒	A description of all covariates tested
☐	☒	A description of any assumptions or corrections, such as tests of normality and adjustment for multiple comparisons
☐	☒	A full description of the statistical parameters including central tendency (e.g. means) or other basic estimates (e.g. regression coefficient) AND variation (e.g. standard deviation) or associated estimates of uncertainty (e.g. confidence intervals)
☒	☐	For null hypothesis testing, the test statistic (e.g. *F*, *t*, *r*) with confidence intervals, effect sizes, degrees of freedom and *P* value noted<br>*Give P values as exact values whenever suitable.*
☒	☐	For Bayesian analysis, information on the choice of priors and Markov chain Monte Carlo settings
☒	☐	For hierarchical and complex designs, identification of the appropriate level for tests and full reporting of outcomes
☐	☒	Estimates of effect sizes (e.g. Cohen's *d*, Pearson's *r*), indicating how they were calculated

Our web collection on statistics for biologists contains articles on many of the points above.

Software and code

Policy information about availability of computer code

Data collection	N/A
Data analysis	We used our homemade open software bcbtoolkit http://toolkit.bcblab.com

For manuscripts utilizing custom algorithms or software that are central to the research but not yet described in published literature, software must be made available to editors/reviewers. We strongly encourage code deposition in a community repository (e.g. GitHub). See the Nature Research guidelines for submitting code & software for further information.

Data

Policy information about availability of data

All manuscripts must include a data availability statement. This statement should provide the following information, where applicable:

- Accession codes, unique identifiers, or web links for publicly available datasets
- A list of figures that have associated raw data
- A description of any restrictions on data availability

Data are available on demand to the authors

Field-specific reporting

Please select the one below that is the best fit for your research. If you are not sure, read the appropriate sections before making your selection.

☒ Life sciences ☐ Behavioural & social sciences ☐ Ecological, evolutionary & environmental sciences

For a reference copy of the document with all sections, see nature.com/documents/nr-reporting-summary-flat.pdf

Life sciences study design

All studies must disclose on these points even when the disclosure is negative.

Sample size	20 participants were analysed in this study.<br>20 is a standard when describing main neuroimaging effects
Data exclusions	no data exclusion
Replication	the effect of interest was also measured at the individual level (i.e. replicated for every subjects)
Randomization	N/A no experimental group per se
Blinding	N/A

Reporting for specific materials, systems and methods

We require information from authors about some types of materials, experimental systems and methods used in many studies. Here, indicate whether each material, system or method listed is relevant to your study. If you are not sure if a list item applies to your research, read the appropriate section before selecting a response.

Materials & experimental systems

n/a	Involved in the study
☒	☐ Antibodies
☒	☐ Eukaryotic cell lines
☒	☐ Palaeontology
☒	☐ Animals and other organisms
☐	☒ Human research participants
☒	☐ Clinical data

Methods

n/a	Involved in the study
☒	☐ ChIP-seq
☒	☐ Flow cytometry
☐	☒ MRI-based neuroimaging

Human research participants

Policy information about studies involving human research participants

Population characteristics	subjects without neurological or psychiatric disease age range 22-42 years
Recruitment	via mailing list.
Ethics oversight	Comité de Protection des Personnes "CPP Ile de France V"

Note that full information on the approval of the study protocol must also be provided in the manuscript.

Magnetic resonance imaging

Experimental design

Design type	resting state
Design specifications	N/A
Behavioral performance measures	N/A

Acquisition

Imaging type(s)	structural - functional - diffusion
Field strength	3T
Sequence & imaging parameters	detailed in full in the manuscript
Area of acquisition	brain

Diffusion MRI ☒ Used ☐ Not used

Parameters: 2 x 60 directions with opposite phase of acquisition

Preprocessing

Preprocessing software	BCBtoolkit
Normalization	diffeormorphic / functional
Normalization template	ICBM152
Noise and artifact removal	ICA-AROMA and Eddy current movement correction
Volume censoring	N/A

Statistical modeling & inference

Model type and settings	Pearson correlation and t-tests
Effect(s) tested	ATLASES

Specify type of analysis: ☐ Whole brain ☐ ROI-based ☒ Both

Anatomical location(s): previously published atlases

Statistic type for inference (See Eklund et al. 2016)	voxel wise
Correction	FWE

Models & analysis

n/a	Involved in the study
☐	☒ Functional and/or effective connectivity
☐	☒ Graph analysis
☐	☐ Multivariate modeling or predictive analysis

Functional and/or effective connectivity	Pearson correlation
Graph analysis	binarized graph, group level degree and betweenness centrality
Multivariate modeling and predictive analysis	we used the method of extraction of the DMN as an independent variable
